# Effects of exercise on cellular and tissue aging

**DOI:** 10.18632/aging.203051

**Published:** 2021-05-13

**Authors:** Priscila Viana Carapeto, Cristina Aguayo-Mazzucato

**Affiliations:** 1 Beta Cell Aging Lab, Joslin Diabetes Center, Harvard Medical School, Boston MA 02115, USA

**Keywords:** exercise, aging, AMPK, type 2 diabetes

## Abstract

The natural aging process is carried out by a progressive loss of homeostasis leading to a functional decline in cells and tissues. The accumulation of these changes stem from a multifactorial process on which both external (environmental and social) and internal (genetic and biological) risk factors contribute to the development of adult chronic diseases, including type 2 diabetes mellitus (T2D). Strategies that can slow cellular aging include changes in diet, lifestyle and drugs that modulate intracellular signaling. Exercise is a promising lifestyle intervention that has shown antiaging effects by extending lifespan and healthspan through decreasing the nine hallmarks of aging and age-associated inflammation. Herein, we review the effects of exercise to attenuate aging from a clinical to a cellular level, listing its effects upon various tissues and systems as well as its capacity to reverse many of the hallmarks of aging. Additionally, we suggest AMPK as a central regulator of the cellular effects of exercise due to its integrative effects in different tissues. These concepts are especially relevant in the setting of T2D, where cellular aging is accelerated and exercise can counteract these effects through the reviewed antiaging mechanisms.

## INTRODUCTION

The natural aging process is carried out by a progressive loss of homeostasis entailing a variety of physiological changes in the function of cells and tissues. To systematically dissect the biological aging process, Lopez-Otin et al. characterized nine major hallmarks of aging that are divided as primary (genomic instability, telomere attrition, epigenetic alterations and loss of proteostasis), antagonistic (deregulated nutrient-sensing, mitochondrial dysfunction and cellular senescence), and integrative hallmarks (stem cell exhaustion and altered intercellular communication). Due to their functional characteristics, primary hallmarks are considered causes of damage, antagonistic hallmarks are responses to damage while the integrative hallmarks reflect the end results of the first two categories [1]. The interconnectivity between the different hallmarks provides a systematic approach to evaluate interventions that target aging at a cellular level.

Exercise is a lifestyle intervention with known antiaging effects capable of counteracting several of the hallmarks of aging including senescence and age-associated inflammation [2–4]. We propose that 5’ adenosine monophosphate-activated protein kinase (AMPK) can orchestrate many of the antiaging effects of exercise through its regulation of diverse cellular pathways in the setting of energetic stress [5]. Activating AMPK is sufficient to extend lifespan in many organisms. It is naturally activated in response to muscle contraction and nutrient depletion, both of which are components of exercise [6]. Whereas most of the studies supporting AMPK as an antiaging strategy are based in animal models, the use of metformin (an AMPK activator) in clinical trials (TAME) as an antiaging drug is based on its capacity to delay heart disease, cancer, cognitive decline and death in people with diabetes [7]. These results suggest that the antiaging effects of AMPK are also relevant in humans, but the molecular mechanisms underlying these effects remain to be determined.

Type 2 diabetes (T2D), a condition that integrates these concepts, is considered a disease of aging, affecting 30 million people in the United States, most of whom are over the age of 50 [8]. Mortality risk is 50% higher in people with T2D with doubled medical costs and lost work and wages per year. Additionally, longevity and healthspan are impaired by its associated health complications including blindness, kidney failure, heart disease, stroke and amputations.

From a pathophysiological point of view, accelerated cellular aging plays a role in T2D. Studies have shown that people with T2D have shorter telomeres and mitochondrial DNA depletion [9] and at a cellular level the following tissues display markers of the hallmarks of aging: endothelium [10, 11], collagen [12], pancreatic β-cells [13] and muscle [14, 15]. Many of these can worsen metabolic control and contribute to the development of cardiovascular complications.

Exercise is known to be an effective lifestyle intervention for T2D since it improves metabolic control. However, to consider the effects of exercise from a cellular aging point of view is a conceptual change in how physical activity is envisioned as a therapeutic tool for diabetes.

Herein, the antiaging effects of exercise are reviewed from a tissue and cellular level, its effects upon the individual hallmarks of aging and how AMPK can integrate many of these effects. Finally, these concepts are applied to the setting of T2D to provide a novel view of how this disease can be approached from a cellular aging perspective.

## Definitions and search criteria

This review follows the guidelines of exercise and physical activity for older adults from the American College of Sports Medicine [16] where exercise is defined as planned, structured and repetitive movement to improve or maintain one or more components of physical fitness. Sedentary living is defined as a way of living or lifestyle that requires minimal physical activity and encourages inactivity through limited choices, disincentives and/or structural or financial barriers.

The aim of the present review paper is to survey the literature related to exercise and its association with longevity and aging. The rationale for conducting this review is that aging is often accompanied by declining cellular homeostasis which is crucial to the development of chronic diseases, but lifestyle interventions can slow down its effects. The literature was surveyed on MEDLINE through freely accessible PubMed as a search engine for the terms: “exercise”, “longevity” and “aging”; the most relevant studies were included as they related to the 9 hallmarks of aging. Additional searches were performed to elucidate the potential role of AMPK activation upon the hallmark of aging. Studies from animal models, human, meta-analysis and bibliographic reviews were consulted and cited accordingly.

## Exercise as an antiaging strategy

The aging process affects longevity and health span which are influenced by both genetic and environmental factors [17]. To systematize its study, Holloszy defined primary and secondary aging. Primary aging refers to the inevitable deterioration of cellular structure and function, independent of disease and environment such as hearing and visual loss. However, secondary aging refers to physiological changes influenced by disease and environmental factors, they are not inevitable and can be accelerated by sedentary lifestyle or delayed by exercise [18]. Examples of secondary aging include insulin resistance, lessened skeletal mass and function, decline of components of the immune system and of cognitive function [19].

The complex relationship between factors that are accelerated by a sedentary lifestyle and those that are solely due to age, has been successfully addressed in various reviews [19, 20]. These studies highlight the importance of studying aging in physically active individuals, ideally in longitudinal studies across the life course of an individual such that the confounding effect of sedentary behavior in the loss of functionality during aging is avoided. As an example, a landmark 21-year longitudinal study at Stanford that followed runners and compared them with a sedentary group, found that those who exercise had a significantly lower risk of dying (15%) during that time frame than the sedentary group (34%) while also having reduced disabilities [21]. It is unclear whether the beneficial effects of exercise in this study were due to a delay in secondary aging or to countering of the effects of sedentarism.

Regardless of this limitation, numerous studies have shown that maintaining a minimum quantity and quality of exercise improves cardiorespiratory fitness and muscle function, flexibility and balance [22]. Current guidelines recommend a minimum of 150 min/week of moderate intensity aerobic activity for maximum longevity benefits, with higher duration and intensity increasing cardiovascular and metabolic effects. It has been estimated that performing three to five times the recommended physical activity (450-750 min/week) reaches the maximal healthspan benefit that can be achieved with endurance exercise. Strength training should be added to minimize loss of muscle mass that is characteristic of aging and disease [23].

The beneficial effects of exercise upon longevity and health span are also evident in individuals that have a genetically determined longevity, such as centenarians. In this unique population, the decline in lung function and sarcopenia can be counteracted by exercise programs which increase their physical capacity and health span [24].

When compared with other interventions directed at slowing aging, such as caloric restriction, some studies have shown that in mice, exercise lacks the adverse outcomes that were observed with time restricted feeding (lean mass and cardiovascular maladaptation) [25] and should therefore be a first line choice as an antiaging strategy. Additionally, it is currently unclear whether caloric restriction has a positive effect in humans. Current research is exploring intermittent fasting as an alternative with beneficial antiaging effects at a cellular level in animals and humans [26, 27].

**Figure 1:**
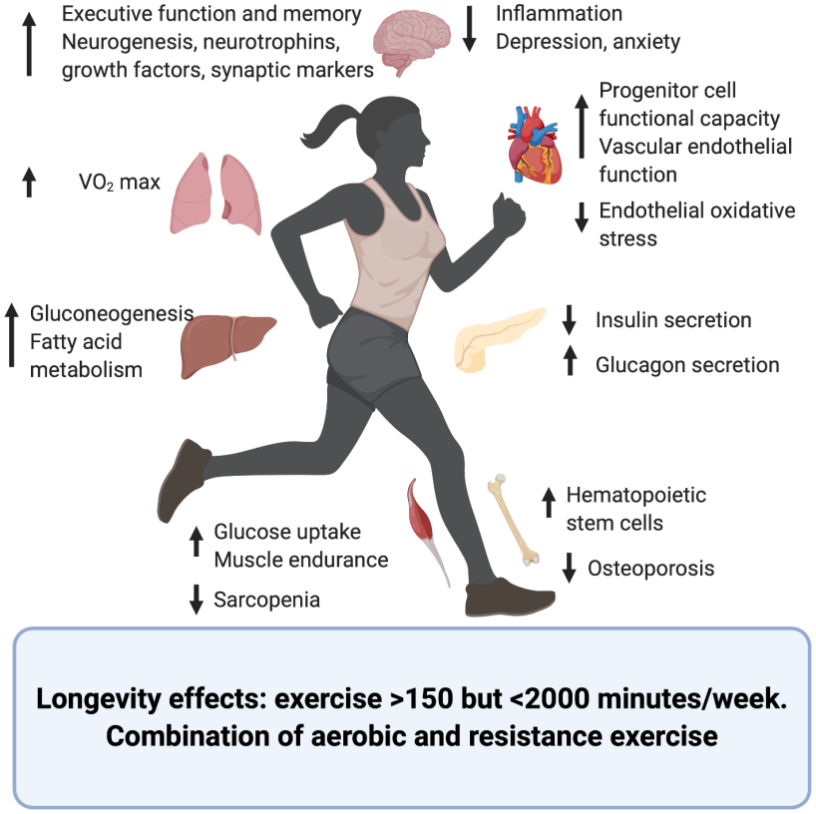
**Effects of exercise upon the aging process of different organs and systems.** Created in BioRender.

The effects of exercise upon different organs and systems and its contribution to longevity and health span have been summarized in Figure 1 and Table 1.

**Table 1:** Effects of exercise on human and animal models of aging.

**Variable (organ or system)**	**Observation**	**References**
Longevity and healthspan	Decreased risk of death	[21, 22]
Cardiopulmonary	Improved maximal oxygen uptake (↑VO_2_ max) Improved atherosclerotic plaque composition (calcification only) Prevention of post-MI complications Improved functional outcome in patients with heart failure with preserved ejection fraction Improved progenitor cell functional capacity Decreased endothelial oxidative stress, improves vascular endothelial function Increased hematopoietic stem cells	[28, 32–37]
Muscle/bone/skin	Prevention of age-associated muscle degeneration Reduced physical disability Reduced sarcopenia Improved muscle endurance Enhanced balance and motor coordination Improved skin structure Increased bone formation, decrease osteoporosis	[45, 47, 48]
Peripheral and central nervous systems	Improved executive function and memory Prevention of Alzheimer disease and other neurodegenerative diseases Improved neurogenesis, neurotrophins, growth factors and synaptic markers Decreased inflammation Restoration of retinal ganglion cells Preservation of neuromuscular junctions Relaxation, decreased anxiety and depression	[49–58]
Metabolism and glucose control	Decrease peripheral insulin resistance Decreased insulin secretion Increased glucagon, gluconeogenesis and fatty acid metabolism Decreased A1c Increased insulin-independent glucose uptake	Reviewed in [62]

### Cardiopulmonary

Cardiovascular (CV) disease is a major cause of mortality worldwide and sedentary lifestyle highly contributes to CV disease burden. Cardiorespiratory fitness, as measured by maximal oxygen uptake (VO_2_ max), is a strong and independent predictor of all-cause mortality [28] and improvement of CV health can be achieved through frequent physical activity and exercise. Even a generally active daily life, without regular exercise, is positively associated with CV health and longevity in older adults [29].

However, appropriate volume and intensity are essential to maximally benefit from exercise interventions as excessive exercise is counteractive [30]. Several publications reviewed in [31] have studied marathon runners as examples of strenuous and endurance exercise. There is general consensus that vigorous exercise, acutely and transiently, increases the risk of sudden cardiac death but only in individuals with underlying cardiac disease. Additionally, several studies have measured cardiac enzymes in runners after completing a marathon and have shown that a subset of them display elevation of cardiac enzyme creatine kinase, troponins and natriuretic peptides, suggesting myocardial injury. Elevation of these markers correlated with younger age, presence of cardiovascular risk factors, running inexperience, increased exercise duration and intensity as well as dehydration. Additionally, it has been shown that prolonged exercise (>2000 min/week) correlates with a higher prevalence of atherosclerotic plaques. However, the composition of these plaques was more benign with fewer mixed plaques and more plaques with only calcification, which might explain the increased longevity of endurance athletes even in the presence of atherosclerotic plaques [32]. Overall, exercise is beneficial to cardiovascular health, and proper training techniques that allow for the proper cardiac adaptations to long-term exercise, also named *athlete’s heart*, can counteract the transient increased CV risk linked to prolonged and strenuous exercise.

The effect of exercise amongst the population with established CV events is also beneficial. After a myocardial infarction, exercise has been shown to prevent future complications, improve the quality of life and longevity of patients [33]. Amongst older adults with heart failure and preserved ejection fraction, exercise training is the most effective intervention to improve functional outcomes. In a mouse model of this disease, RNASeq of explanted hearts showed that exercise reversed age-related pathways such as those that correlated with cell cycle [34].

One of the mechanisms by which exercise mediates CV benefits is by enhancing the function of endothelial progenitor cells, which play a role repairing endothelial injuries. With age, progenitor cells have been shown to dysfunction; exercise increases expression of CXCR4 and phosphorylation of JAK-2 thus improving progenitor cell functional capacity [35]. Additionally, exercise decreases age-associated vascular endothelial oxidative stress, improves vascular endothelial function [36] and increases hematopoietic stem cells, markers of neovascularization and vascular repair [37].

### Muscle/bone/skin

Loss of muscle mass is characteristic of aging and it starts to decline after 25-30 years of age such that by 80 years 40% of muscle mass has been lost [38, 39]. This is thought to contribute to a wide array of age associated pathologies such as frailty, weakness, loss of function, metabolic syndrome, cancer, Alzheimer’s and Parkinson’s disease [39–41], and is believed to be secondary to the loss of myokines (muscle-derived growth factors and cytokines) that modulate systemic physiology.

Progressive skeletal muscle wasting is known as sarcopenia and is characterized by a decrease in muscle cross-sectional area due to reduction fiber number and its atrophy [42]. A number of mechanisms underlying this process have been proposed, being correlated with the primary and antagonistic hallmarks of aging, including loss of mitochondrial density and instability of its DNA (mtDNA) [43]. Another aspect suspected to play a role is failure of adaptive responses to contractile activity, such as the ability to clear reactive oxygen species. A study using mice lacking the Cu, Zn superoxide dismutase showed an accelerated, age-related loss of muscle mass and function which correlated to chronic exposure to increased oxidant activity [44].

Regular physical activity is the only efficient intervention to prevent and treat this age-associated degeneration. Aerobic endurance training improves peak oxygen consumption by 10-15% while resistance training increases muscle strength and mass. On a mechanistic level, exercise reduces sarcopenia by decreasing inflammation and increasing anabolism and protein synthesis [45]. Additionally, activation of peroxisome proliferator-activated receptor gamma (PGC-1α) improves muscle endurance, mitochondrial remodeling and enhanced balance and motor coordination in animal models [46].

Bone is another tissue profoundly affected by secondary aging. Loss of bone mass and strength characterize the aging process predisposing to the onset of osteoporosis and fractures. Exercise interventions are a long-term strategy to maximize bone mass and delay the onset of osteoporosis. These interventions need to include weight-bearing activities to generate bone formation and delay telomere shortening and modification of DNA methylation [47].

Skin, a component of the integumentary system, is also affected by secondary aging which deteriorates its structure compromising its function as a barrier, healing and making it prone to disease. Endurance exercise attenuates age-associated changes to skin in humans and mice partly through IL15, which acts as a regulator of mitochondrial function in aging skin. Upregulation of IL15 is thought to occur through activation of muscle AMPK, a central regulator of metabolism, therefore the elimination of muscle AMPK causes a deterioration of skin structure [48].

### Peripheral and central nervous systems

Chronological aging is associated with a decline in cognitive, memory and executive functions as well as a decline in peripheral nervous system such as neuromuscular junctions. Some of these changes are thought to be due to primary aging and are therefore not amenable to interventions; however, a subset of age-related changes is thought to be due to secondary aging and therefore is influenced by sedentary lifestyle and exercise.

High levels of exercise have been associated with better executive function and memory in cross-sectional and longitudinal analyses [49]. In fact, regular physical activity is one of the few interventions capable of preventing Alzheimer’s disease and other age-associated neurodegenerative disorders [50]. This benefit relates to exercise’s ability to increase the endurance of cells and tissues to oxidative stress, and to increase vascularization, energy metabolism and neurotrophin synthesis, all of which play a role in neurogenesis, memory and brain plasticity. Additional mechanisms of exercise action on the central nervous system are increased neurotrophins, growth factors and synaptic markers coupled with a reduction in inflammation [51–55].

Retinal ganglion cells (RGCs) which become vulnerable to injury with advancing age with resultant impaired vision, are also restored by exercise in mice. This is due to sustained levels of brain-derived neurotrophic factor (BDNF) levels in the retina underscoring the role of this critical factor in maintaining retinal health during aging of animal models [56].

Within the peripheral nervous system, neuromuscular junctions modify their structure with age. These changes are characterized by axonal swellings, sprouting, synaptic detachment, partial or complete withdrawal of axons from some postsynaptic sites and fragmentation of the postsynaptic specialization. However, one month of voluntary exercise in 22-mo-old mice reversed age-related synaptic changes with no change on motor neuron number or muscle fiber turnover [57].

Additionally, the psychological effects of exercise are profound and include relaxation and alleviation of anxiety and depression. These effects are strong enough that exercise can turn into an addiction [58]. Given its effectiveness and safety, it should be considered a first line of choice to treat many psychological ailments among the elderly, including insomnia.

### Metabolism and glucose control

Secondary aging is associated with the development of insulin resistance, increased adiposity, and accumulation of ectopic lipid deposits in tissues and organs; all of which contribute to metabolic dysfunction [59] increasing the risk of T2D.

Several metabolic alterations accumulate over time along with a reduction in physical fitness, suggesting the existence of a "metabolic clock" that influences aging. The main features of the "westernized" lifestyle (hypercaloric nutrition and sedentary behavior) accelerate the metabolic decline of secondary aging factors, such as insulin resistance, while the promotion of metabolic fitness leads to health span extension [60].

The beneficial effects of exercise upon glucose metabolism are well known and have been thoroughly studied [61], converting increase physical activity in one of the pillars of the treatment of T2D. The human body reacts to an acute bout of exercise by decreasing insulin secretion and increasing circulating glucagon, leading to improved insulin sensitivity and decreased glycosylated hemoglobin [62]. However, exercise as an antiaging strategy in the context of T2D is novel and could add to its known beneficial metabolic effects.

In summary, exercise has shown to have beneficial antiaging effects of many human organs and tissues either by reversing some of the aging phenotypes or by delaying their appearance (Figure 1 and Table 1).

## Signaling pathways through which exercise mediates anti-aging effects

The consequences of exercise on the aging of specific organs and tissues can be studied at a cellular perspective and structured based on the changes it has upon the hallmarks of aging.

### Primary hallmarks

### Genomic instability

Age is characterized by the accumulation of lesions in the DNA and defects in the nuclear architecture leading to genomic instability. These are the result of exogenous (physical, chemical and biological agents) and endogenous factors (DNA replication errors, spontaneous hydrolytic reactions and reactive oxygen species) [63] that result in mutations, translocations, chromosomal gain and losses, telomere shortening and gene disruption.

Exercise minimizes these lesions, partly through: reduction of the age-associated 8-hydroxy-2'-deoxyguanosine (8-OHdG) [64], increased activity of DNA repair, resistance to oxidative stress in proteins, and nuclear factor kappa B (NF-kB) and PGC-1α signaling [64–66].

### Telomere attrition

Telomeres protect the integrity of chromosomal DNA during cellular division but are particularly susceptible to age-related deterioration [67]. Studies have demonstrated a direct correlation between telomere length and life expectancy, stress, DNA damage and onset of age-related diseases. Various genetic and environmental factors, such as diet, physical activity, obesity and stress, are known to influence health and longevity as well as telomere dynamics.

Exercise is able to increase telomere length through changes in telomerase activity, inflammation, oxidative stress and skeletal muscle satellite cell content. Long-term exercise can activate telomerase reverse transcriptase (TERT) in leukocytes and also upregulate protective and DNA repair regulator proteins (such as telomeric repeat-binding factor 2 and Ku protein). This has important physiological consequences since a positive correlation has been shown between muscle regeneration processes and telomere length in older adults [68].

### Epigenetic alterations

Exercise is capable of inducing widespread epigenetic changes. General loss of histones, imbalanced histone modifications, transcriptional deregulations, changes in heterochromatin, breakdown of nuclear lamina, as well as DNA and histone methylation, are characteristics of aging [69].

Physical activity increases DNA methylation, causes histone modifications and induces miRNA in muscle, brain and the cardiovascular system. Acute aerobic exercise decreases methylation of PGC-1α, mitochondrial transcription factor (TFAM), MEF2A, citrate synthase (CS) and pyruvate hydrogenase kinase isozyme (PDK4) [70]. In addition, aerobic-induced SIRT-1 downregulates p53, PGC-1α and NF-kB via its deacetylase activity [71, 72]. Chronic moderate aerobic exercise reduces inflammation through a decrease of pro-inflammatory cytokines (IL-1b and IL18) that is mediated by methylation of pro-inflammatory apoptosis-associated speck-like protein caspase (ASC) gene [73].

### Loss of proteostasis

Some age-related diseases are linked to impaired protein homeostasis – known as proteostasis. Cell autophagy is one of the mechanisms for degradation and recycling of damaged macromolecules and organelles, and its alteration can lead to disease. Although human data are still scarce, muscle autophagy markers are up-regulated after exercise training in older women [74] and could underlie the promotion of health span and longevity.

The target of rapamycin complex 1 (TORC1) - a central kinase involved in protein translation- is a negative regulator of autophagy and so may be an effector of exercise. TORC-1 is downregulated by exercise through modulation of IGF-1, Akt/mTOR, and Akt/FoxO3a signaling. This cascade has been shown to prevent loss of muscle mass and strength [75, 76]. Additionally, the protective effect of chronic exercise on diabetes-induced muscle atrophy is partly due to decreased muscle autophagy [77].

### Antagonistic hallmarks

### Deregulated nutrient-sensing

Deregulation of nutrient sensing pathways have been extensively involved in age-related phenotypes, and their downregulation is one of the most effective strategies to extend lifespan and health span. As humans age, the loss of muscle mass occurs due to acute changes in net protein balance, particularly in the myofibrillar protein fraction [78, 79], and exercise regulates the nutrient sensing pathways.

Insulin like growth factor (IGF-1) acts as a key link between mechanical contraction and protein synthesis since it is acutely stimulated and promotes ribosomal biogenesis and translation to form new myofibril proteins. During exercise the mechanical loading and contraction cause the local release of IGF1 which activates IGF and leads to to muscle protein synthesis [80].

**Figure 2:**
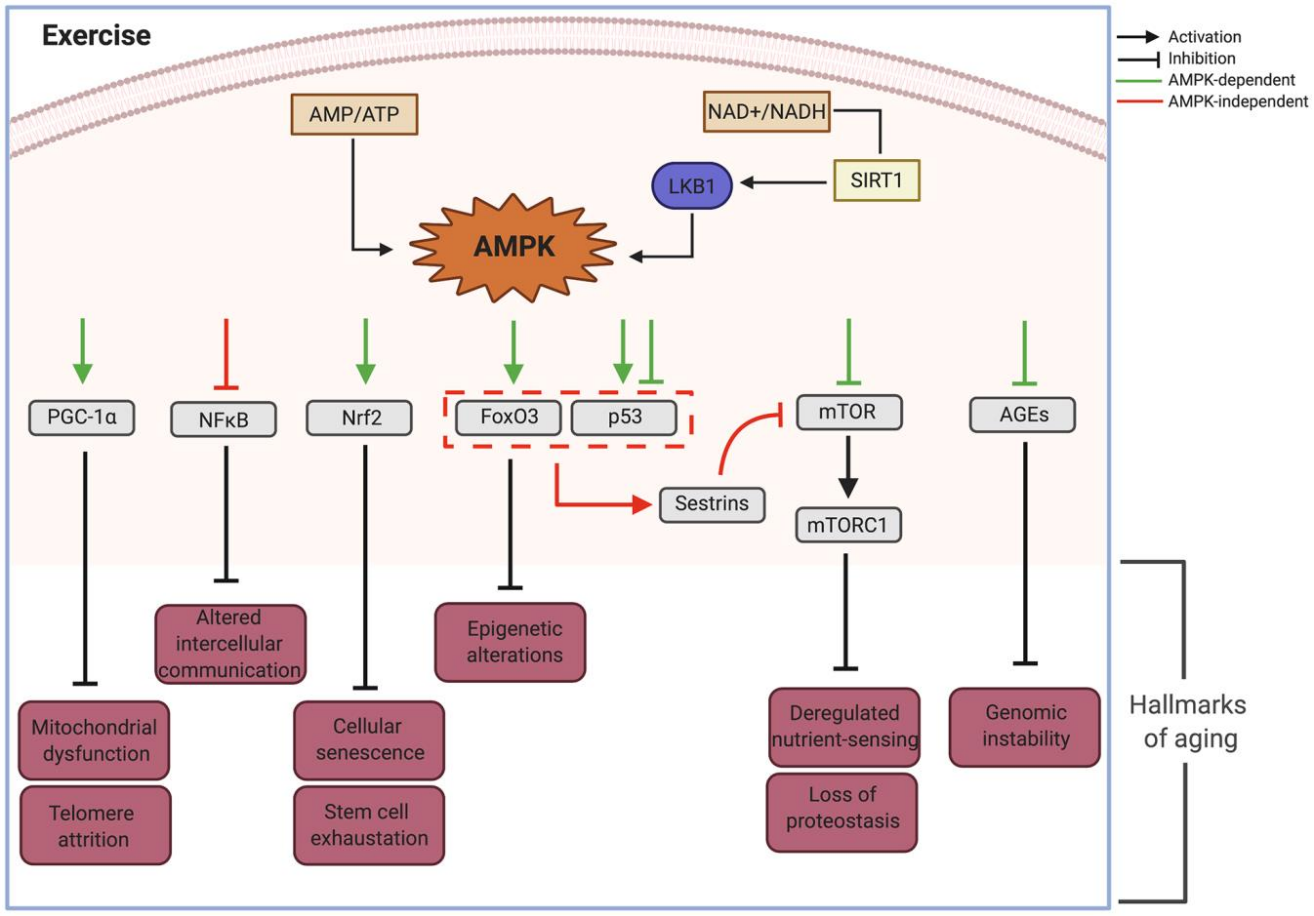
**AMPK as an effector node on the effects of exercise upon the different hallmarks of aging.** AMP, adenosine monophosphate; AMPK, AMP- activated protein kinase; ATP, adenosine triphosphate; AGEs, advanced glycation end-products; FoxO3, Forkhead Box O3; LKB1, Liver kinase B1; mTOR, mammalian target of rapamycin; mTORC1, mTOR complex 1; NAD+, Nicotinamide adenine dinucleotide; NADH, Reduced Nicotinamide adenine dinucleotide; NFkB, Nuclear Factor kappa-light-chain-enhancer of activated B cells; NRF2, Nuclear factor erythroid 2-Related Factor 2; p53, Tumor suppressor protein 53; PGC-1, peroxisome proliferator-activated receptor gamma; SIRT1, Silent information regulator. Created in BioRender.

Another exercise-regulated nutrient sensing pathway is AMPK, which is activated in response to decreased intracellular ATP and changes in the NAD+/NADH ratio. Its function is to preserve ATP by inhibiting both biosynthetic and anabolic pathways while simultaneously stimulating catabolic pathways to re-establish cellular energy stores. The increased concentration of Ca^2+^ during muscle contraction can also directly activate AMPK and is implicated in the regulation of numerous intracellular proteins that mediate cellular transduction, including kinase C, calcineurin, and CaMKs [81–83]. Both AMPK and CaMKII lead to PGC-1α activation, a member of a family of transcriptional coactivators that regulate mitochondrial biogenesis [84] (Figure 2).

Oxidative stress is yet another mechanism through which exercise can regulate nutrient sensing by producing sestrins and activating the MAPK cascade [85], a family of intracellular signaling that include the extracellular signal regulated kinase 1 and 2 (ERK1/2), the c-Jun NH2- terminal kinase (JNK) and p38 [86]. Activation of this pathway leads to the inhibition mTOR complex 1 (mTORC1) [87] and activation of PGC-1α [88].

### Mitochondrial dysfunction

The accumulation of mitochondrial damage due to ROS generated from the electron transport chain is the base of the mitochondrial theory of aging first proposed by Harman [89]. It postulates that the oxidative damage to mtDNA affects cellular replication and transcription, altering the functionality of mitochondrial proteins.

It has been known for a long time that exercise increases mitochondrial content in skeletal muscle [90]. Additionally, it can attenuate mitochondrial dysfunction through recovery of oxidative capacity and the activity of electron transport chain protein complexes [90, 91]. In agreement with this, endurance athletes showed absence of age-related decline in mitochondrial oxidative capacity and elevated expression of mitochondrial proteins, mtDNA and mitochondrial transcription factors [92]. In mtDNA mutator mice, which exhibit an accelerated aging phenotype, a 5-month aerobic exercise program promoted systemic mitochondrial biogenesis, prevented mtDNA depletion and mutations, increased mitochondrial oxidative capacity and respiratory chain assembly. These changes restored mitochondrial morphology and blunted pathological levels of apoptosis in multiple tissues [93].

Regular exercise has a profound beneficial effect on human mitochondrial function and biogenesis, partly mediated by PGC-1α upregulation. Phosphorylation of PGC-1α drives the production of fibronectin type III domain-containing protein 5 (FNDC5), followed by its cleavage to generate irisin [94–96], which can be secreted, activated and transported to multiple tissues to exert its beneficial effects.

### Cellular senescence

Cellular senescence is characterized by lack of cellular proliferation in response to stressors and secretion of an array of proteins specific to each cell type. This array of proteins known as senescence-associated secretory phenotype (SASP) is part of the aging hallmark of altered intercellular communication.

Cellular senescence is linked to other mechanisms of aging. ROS accumulation in mitochondria leads to single-strand DNA breaks that accumulate in telomere regions and result in telomere shortening and premature cellular senescence. Senescence has been linked to numerous age-related chronic diseases and risk factors, such as T2D [13, 97, 98]. Exercise enhances telomere length and reduces the expression of apoptosis regulators (such as cell cycle checkpoint kinase 2, p16^INK4a^, and P53) shedding light on the beneficial impact of exercise on senescence [68].

Expression of p16^INK4a^, a marker and effector of senescence, in cellular fractions of human whole blood exponentially increased with chronological age and associated significantly with sedentary life style [99]. In addition, p16^INK4a^ expression correlated with plasma interleukin-6 (IL-6) concentration, a marker of human frailty. Exercise induces increased IL-6 derived from muscle which has anti-inflammatory properties, whereas paradoxically, IL-6 resulting from TNF or NFkB activation relates to aging phenotypes [100]. In a senescence rat model, exercise suppresses senescence markers and down-regulates inflammatory mediators by reducing gamma glutamyltranspeptidase activity and levels of p53, p21, and IL-6 [101].

In some settings, exercise-induced senescence is beneficial, such as the appearance of fibro-adipogenic progenitors in response to muscle damage, and leads to regenerative inflammation [102].

### Integrative hallmarks

### Stem cell exhaustion

A decline in the regenerative potential of tissues is expected with age. Specifically, a decline in satellite cells results in impaired repair of muscle fibers while decreased hematopoietic stem cells leads to immunosenescence [103–105]. Exercise is one of the most potent stimuli for the migration of stem cell subsets from their home tissue to impaired ones for later regeneration. It increases the number and differentiation of satellite cells type II fibers [106]. In addition, exercise activates pluripotent cell progenitors in several tissues, including mesenchymal and neural stem cells leading to improved brain regenerative capacity and cognitive ability [107].

### Altered intercellular communication

Pro-inflammatory tissue, damage accumulation, cumulative dysfunction of the immune system and elevated levels of pro-inflammatory cytokines secretion underlie the development of inflammaging, a pro-inflammatory phenotype associated with progressive aging that affects intercellular communication [108]. This is characterized by the activation of the NOD-like receptor protein 3 (NLRP3) and elevation of IL-1b, tumor necrosis factor-a (TNF-α) and interferons [108, 109]. Exercise downregulates this inflammatory response through AUF1 [110], a decay factor implicated in maintenance of telomere length by TERT modulation [111].

Moreover, exercise further suppresses inflammation via IL-6 released from muscle [112]. Recent studies support the notion that IL-6 can activate pathways that have insulin-sensitizing effects [113, 114] by activating AMPK in skeletal muscle, leading to increased glucose uptake and translocation of the glucose transporter GLUT4 from intracellular compartments to the plasma membrane [115]. Chronic moderate exercise increases methylation levels of the pro-inflammatory apoptosis-associated speck-like protein caspase (ASC) gene that controls secretion of IL-1β and IL-18 in leukocytes [73]. Exercise also controls age-related increases of pro-inflammatory cytokines thereby preventing accumulation of misfolded proteins [1, 116].

In summary, exercise attenuates all hallmarks of aging through different molecular pathways and effectors that seem independent and disconnected. We hypothesize there must be molecular regulatory nodes able to coordinate these responses and that AMPK can play such a role.

## AMPK as a central regulator

We propose that activation of AMPK plays a significant integrative role impacting the primary, secondary and integrative hallmarks of aging in response to exercise. In muscle, AMPK is a long known exercise effector that is activated by increased AMP/ATP and NAD^+^/NADH ratio [117]. Mammalian AMPK is a heterotrimeric complex with α, β, and γ subunits. Mechanistically, AMP interacts with AMPK’s γ subunit, facilitating activation of the α subunit by upstream regulatory kinases such as LKB1. In parallel, the increase in NAD^+^/NADH causes activation of silent information regulator 1 (SIRT1) deacetylase activating LKB1.

Thus cellular energy balance effectively controls cellular responses via an integrated signaling network mediated by AMPK [118], which phosphorylates its downstream targets and is able to attenuate the hallmarks of aging [119, 120] (Figure 2). Below is a list of the main effectors of AMPK and their actions upon the hallmarks of aging.

### PGC-1α

PGC-1α is a critical regulator of gene transcription that controls energy homeostasis and is involved in mitochondrial biology [121]. In mouse skeletal muscle cells, PGC-1α mediates the conversion of IIb fibers into mitochondria-rich type IIa and I fibers [122]. Although PGC-1α mediated conversion has not been directly shown across species, type IIa fibers in humans have the highest concentration of PGC-1a [123, 124], which could support a parallel mechanism. In addition, PGC-1α activation by AMPK has shown to act as a regulator of human telomere transcription via telomeric repeat-containing RNA (TERRA), important for telomere integrity [125].

Some of the effects of PGC-1α are mediated through its inhibition of NFκB. Ablation of PGC-1α led to activation of NFκB and upregulated pro-inflammatory cytokines [126] while increased expression of PGC-1α inhibited NFκB signaling in aortic smooth muscle and endothelial cells [127]. These observations suggest that AMPK controls NFκB activation and that deficiency of AMPK signaling during aging disturbs energy metabolism and enhances inflammation (Figure 2).

### Nuclear factor erythroid 2-related factor 2 (Nrf2)

Nrf2 is a basic leucine zipper protein involved in regulation of antioxidant proteins that protect against oxidative damage triggered by injury and inflammation. Studies using AICAR (an AMP analog and AMPK activator) showed stimulated expression of Nrf2 and upregulation of glutathione peroxidase 7 (Gpx7), leading to suppression of cellular senescence and SASP [128]. In addition, activation of Gpx7 via Nrf2 delayed cellular attrition related to stem cell aging [128, 129] (Figure 2).

Crosstalk between AMPK activated signaling pathways is demonstrated by inhibition of Nrf2 by the p65 component of NFκB complex through kelch-like ECH-associated protein 1 (Keap1). This crosstalk can have an additive effect upon decreased cellular senescence and stem cell maintenance (Figure 2).

### FoxO3a

Activation of the FoxO3a axis by AMPK increased stress resistance in long-lived animals [118]. By mediating epigenetic and transcriptional changes, FoxO3, a member of the FOXO subfamily of forkhead transcription factors, is able to mediate the effects of therapeutic interventions on age-related diseases and promote healthy aging [120]. Target genes of the AMPK-FoxO3 pathway include uncoupling protein UCP2 and GAD45a, which are involved in defense against oxidative stress and DNA damage leading to longevity [130] (Figure 2).

### P53

Tumor protein P53 regulates the cell cycle and functions as a tumor suppressor. The effects of exercise-activated AMPK upon P53 are complex, with both activating and inhibiting effects. AMPK activation has been shown to induce phosphorylation of P53 and lead to cell cycle arrest. This promotes cellular survival in response to glucose deprivation (as might occur during exercise), however these cells can rapidly reenter the cell cycle upon glucose restoration. However, persistent activation of AMPK leads to accelerated P53-dependent cellular senescence, underscoring the importance of the timing and pulsatility of AMPK activation [131]. Interestingly, acute exercise has also been shown to decrease nuclear P53 directly or through upregulation of Nrf2 leading to inactivation of P53-P21^Cip1^ and P16^INK4a^-RB signaling pathways [118, 132]. Due to these varied effects *in vitro*, it has been difficult to elucidate the *in vivo* functional role of P53 during aging. It is likely that the response partly depends on its cellular localization as well as the duration and intensity of the stimulus.

### FoxO and P53

When activated simultaneously by AMPK, P53 and FoxO can induce the expression of sestrins, a family of highly conserved stress-response proteins with oxidoreductase activity that can protect cells from oxidative stress. Loss of sestrins has been linked to age related pathologies such as mitochondrial dysfunction, muscle degeneration and lipid accumulation. These effects are attributed to increased TOR activity and the associated decrease in autophagic uptake (Figure 2). These pathologies were prevented by the activation of AMPK by AICAR and the inhibition of TOR by rapamycin [133]. Thus, sestrins are suggested as part of a negative feedback loop through mTOR signaling that operates via the activation of AMPK [118].

### mTOR

Serine/threonine protein kinase mTOR was identified in mammalian cells as a target of the antiproliferative molecule rapamycin [134]. It participates in the formation of two protein complexes called mTORC1 and mTORC2, known be sensitive and insensitive to rapamycin, respectively [135]. Phosphorylation and activation of AMPK leads to inhibition of mTORC1 through v-ATP-ase-AXIN/LKB1, which leads to increased lifespan in *C. elegans* [136].

Whereas autophagy declines during aging, AMPK activation can restore it by inducing the dissociation of mTORC1 from the ULK1 complex, directly binding and phosphorylating ULK1, an autophagy-initiating kinase, with the result of stimulating autophagy. [137]. Furthermore, the direct inhibition of mTORC1 by AMPK can have effects similar to nutrient depletion [120] (Figure 2).

Autophagy and protein synthesis inhibition mediated by downregulation of mTOR have direct effects on proteostasis. In a mouse model of Parkinson’s disease, AMPK activation reversed behavioral impairments, reduced α-synuclein accumulation and enhanced LC3-II-mediated autophagy in dopaminergic neurons [138, 139]. AMPK-activation has also been shown to rescue misfolding and trafficking of rhodopsin, highlighting the AMPK role against impairment in proteostasis [140] (Figure 2).

### Advanced glycation end products (AGEs)

AMPK-activation can also exert antiaging effects through inhibiting the effects of AGEs [141]. AGEs, major inflammatory mediators in macrophages, affect the progression of age-related atherosclerosis and diabetes and inhibit AMPK activity through allosteric competitive binding to its AMP-binding site in the γ subunit [142]. However, AMPK activation inhibits AGEs-induced inflammatory response in murine macrophages [143]. These findings show bidirectional modulation between these two pathways that can be shifted through environmental factors to enhance protective mechanisms against genotoxic stress.

In summary, AMPK activation through exercise can impact all the hallmarks of aging through different signaling pathways as summarized in Figure 2 and can act as a signaling node capable of orchestrating many of the effects of exercise on the health span of different tissues and organs.

## Effects of exercise on cellular aging in T2D

T2D is a complex disorder that combines a genetic hereditary component and environmental risk factors, such as nutrition and lifestyle. Amongst the risk factors, age stands out with most patients being over 60 years old. There is evidence of accelerated cellular aging with hyperglycemia and in both Type 1 (T1D) and Type 2 (T2D) diabetes mellitus [11, 144].

Hyperglycemia increases the hallmarks of aging, such as senescence of endothelial cells in atherosclerotic lesions and telomere shortening [10]. The exposure of endothelial progenitor cells to high glucose concentrations increased cellular senescence in aortas of a streptozotocin-induced diabetes model [11], strengthening the association among hyperglycemia, diabetes and senescence [10]. Additionally, increased mitochondrial DNA depletion and increased aging of collagen have also been reported in patients with T2D [9, 12].

Pancreatic β-cells, which play a crucial role in the development of T2D, have also been shown to undergo cellular senescence in the setting of insulin resistance [145], T2D and high body mass index (BMI). Senolysis (the specific removal of senescence cells either pharmacologically or through transgenic models) improved insulin secretion, blood glucose levels and the gene identity of the remaining β-cell population [13]. Muscle is another tissue impacted by accelerated aging during diabetes as evidenced by accelerated loss of strength and mitochondrial dysfunction in T1D [14, 15]. Skin biopsies obtained from subjects of different ages demonstrated that the onset of cellular senescence occurred earlier in people with juvenile diabetes and in subjects genetically predisposed to diabetes [144]. Additionally, premature senescence has been observed in endothelial colony-forming cells in the cord blood of infants from mothers with diabetes [146]. These data suggest T2D as a disease where cellular aging is accelerated, and therefore is a pathology in need of strategies that can broadly impact aging at the molecular level.

Exercise and physical activity are already cornerstones in the metabolic management of T2D [147]. Randomized trials have shown that lifestyle interventions including 150 minute of physical activity per week, combined with diet-induced weight loss, reduced the risk of T2D by 58% in an at-risk population [148, 149]. Increasing physical activity in adults with T2D resulted in complete remission of the disease in 11.5% of subjects within the first year of intervention and an additional 7% had partial or complete remission of type 2 diabetes after 4 years [150].

**Figure 3:**
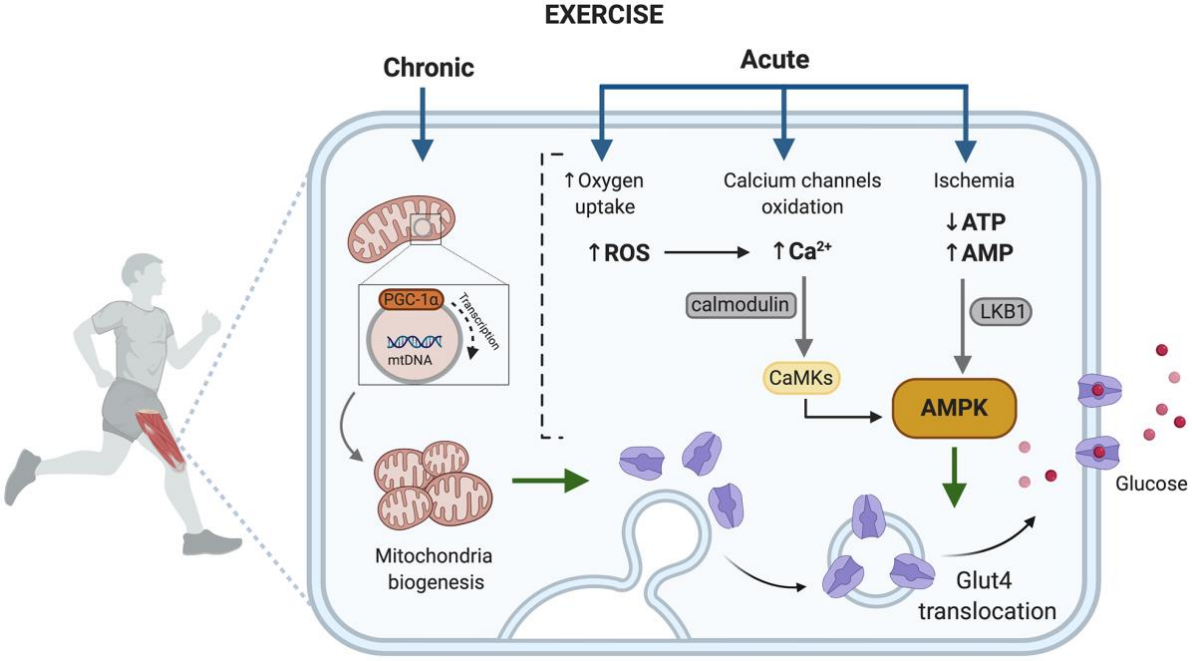
**Exercise activated pathways in muscle capable of contributing to improved metabolic control in T2D.** AMP, adenosine monophosphate; AMPK, AMP- activated protein kinase; ATP, adenosine triphosphate; Ca2+, divalent cation calcium; CaMKs, calcium/calmodulin dependent protein kinases; GLUT4, glucose transporter type 4; LKB1, liver kinase B1; PGC-1, peroxisome proliferator-activated receptor gamma; ROS, reactive oxygen species. Created in BioRender.

The benefits are mainly related to exercise improving blood glucose levels through both a reduction of peripheral insulin resistance [151–153] and its capacity to induce insulin-independent glucose uptake [154]. Depending on whether the exercise is acute or chronic, the activated pathways in muscle are different (Figure 3). Acute exercise is able to promote translocation of GLUT4 to the plasma membrane through at least two signaling pathways, one involves AMPK and the second an increase of intracellular Ca^2+^. Muscle cell contraction is ATP-dependent and an acute exercise bout increases AMP levels, activating the AMPK signaling pathway and leading the fusion of GLUT-4 containing vesicles with the plasma membrane [155, 156] (Figure 3).

Muscle contraction increases ROS generation due to high oxygen consumption that takes place during mitochondrial activity, in fact, superoxide generation in skeletal muscle increases about 50-100-fold during exercise [157, 158]. ROS have been reported to inhibit plasma membrane Ca^2+^ATPase activity indirectly by formation of reactive aldehydes. Hence, ROS would hinder Ca^2+^ removal from the cell and encourage intracellular Ca^+2^ accumulation. Muscle contraction can also directly increase intracellular Ca^2+^, which promotes the membrane translocation of GLUT-4 [81].

Chronic exercise increases the number and activity of mitochondria in muscle [159], which counteracts the reported decrease in size, function and integrity of mitochondria in people with T2D [160], and decreases the expression of PGC-1α, a marker of mitochondrial biogenesis [161]. Furthermore, skeletal muscle mitochondrial dysfunction has been linked with insulin resistance and can have implications on inflammation, senescence, autophagy and retrograde nuclear signaling [162].

In summary, exercise activates molecular signals that can bypass defects in insulin signaling in skeletal muscle and increase skeletal muscle mitochondria, which are associated with improved insulin sensitivity in skeletal muscle and therefore improve aging-associated effects of T2D.

## Summary, perspective and limitations

**Figure 4:**
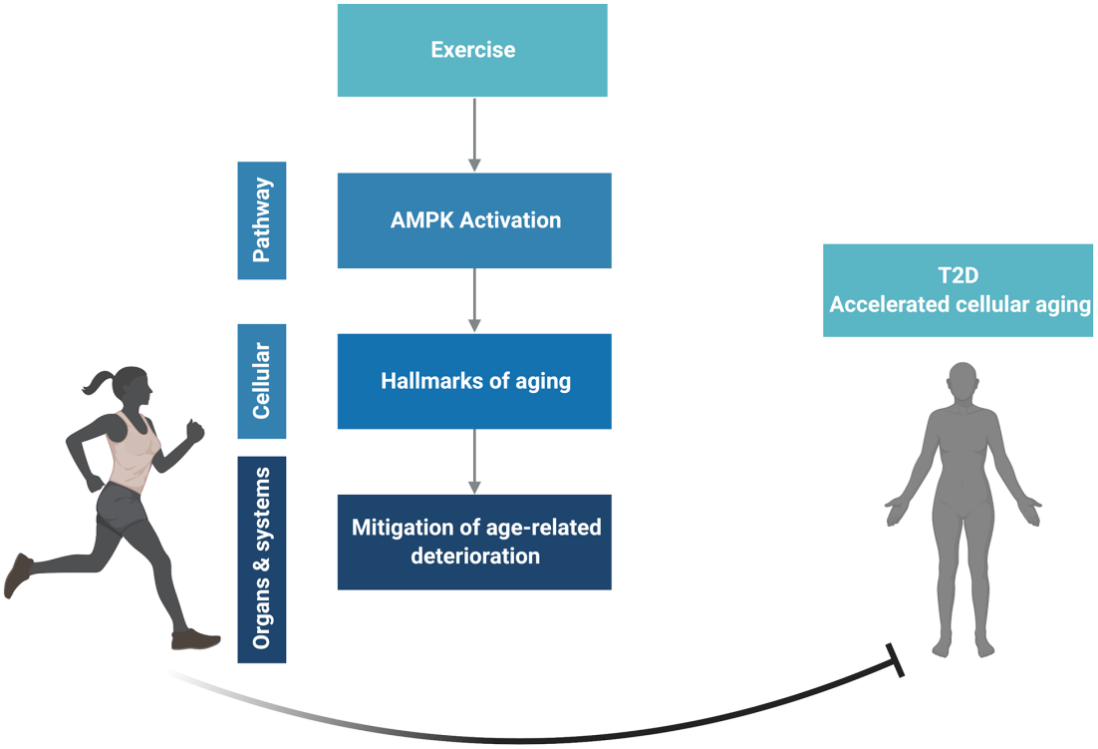
**Conceptual overview.** Created in BioRender.

Exercise is an effective strategy to prevent aging and enhance longevity and health span both on a clinical and a cellular level due to its capacity to modulate all nine hallmarks of aging. Additionally muscle, one of the main systemic effectors of exercise, is recognized as an endocrine organ that produces and releases myokines, implying a complex cross talk between muscles and other tissues. The AMPK pathway (Figure 2), a well-known mediator of exercise effects in muscle could be activated in different tissues and drive many of the health-promoting and lifespan-extending capabilities of exercise. We propose that it is a central effector node able to impact the hallmarks of aging and integrate the effects of exercise on many tissues. T2D, a disease in which cellular aging is accelerated in several tissues, is an ideal candidate to further understand the antiaging effects of exercise (Figure 4).

This review has several limitations. As mentioned, the lack of deleterious effects of a sedentary lifestyle upon aging during exercise can sometimes be confused with antiaging effects of exercise. This conundrum can only be solved if aging studies are carried out in non-sedentary older populations. Unfortunately this rarely occurs and should be considered while interpreting the cited studies. Another limitation is the cross-sectional design of studies comparing an exercised and a sedentary population in spite of the knowledge that the rate of aging varies considerably amongst individuals. The ideal design for aging studies is a longitudinal follow up of non-sedentary individuals; this is rarely feasible due to constraints of time and resources.

Although every attempt was made to include the most relevant studies for each subject, the vastness of publications in this area means that some important work may have been unintentionally omitted. We encourage readers to further their searches on specific subjects that have specially interested them.

We propose that future studies should address the effects of exercise on tissues which are not considered its direct targets but do show accelerated aging in T2D, such as pancreatic β-cells. In these, the role of AMPK and its physiological control will become especially significant as exercise is considered a cellular antiaging strategy.
